# Observations on White Grubs Affecting Sugar Cane at the Juba Sugar Project, South-Western Somalia, in the 1980s, and Implications for Their Management

**DOI:** 10.3390/insects4020241

**Published:** 2013-06-18

**Authors:** Matthew J. W. Cock, Gillian B. Allard

**Affiliations:** CABI, Bakeham Lane, Egham, TW20 9TY, UK

**Keywords:** Cochliotis melolonthoides, Brachylepis werneri, Schizonycha, Ophiocordyceps barnesii

## Abstract

The authors made two visits to the Juba Sugar Project in south-west Somalia, at the beginning of the minor rains in October 1986, and at the beginning of the main rains in March 1987. Observations were made on morphospecies of scarabaeid white grub larvae, the adults, and the two associated for the key economic species, *Cochliotis melolonthoides* and *Brachylepis werneri*. Sampling larvae and adults by digging soil quadrats and adults by light trapping gave useful information on their biology and phenology. Sampling methods were evaluated and economic thresholds were extrapolated based on earlier work. Natural enemies were surveyed, and entomopathogenic nematodes and a cordyceps fungus (*Ophiocordyceps barnesii*) were considered to have potential to be used as biological control interventions.

## 1. Introduction

White grubs are the larvae of those scarabaeid beetles which feed below ground on plants roots. Although white grubs belong to the family Scarabaeidae, not all scarabaeids are white grubs—other groups such as dung beetles are also included in the family. Wilson [1] provided a global review of what is known about white grub pests of sugar cane and their natural enemies, which is still valid. Wilson [1] points out that Scarabaeidae is divided into several subfamilies, and white grubs occur in at least three of these: Melolonthinae, Rutelinae and Dynastinae. Dynastinae white grubs differ from the others in that the adults also feed on the cane and can cause very significant damage to young growth. The life cycle of a typical melolonthine white grub takes one year, although some species are known to take longer [1].

The literature on sugar cane white grubs in Africa is limited, but it shows that the species of white grubs which cause damage to sugar cane vary from country to country. Box [2] lists *Schizonycha vastatrix* Chiaramonte (Melolonthinae) and *Heteronychus paolii* Arrow (Dynastinae) as sugar cane pests in Somalia. *Heteronychus paolii* is a replacement name for *H. sacchari* Paoli which was described from Somalia, the adults attacking sugar cane below the surface of the ground [3,4]; it may well be a synonym of *H. abyssinica* [5]. There seems to be no other information available on the white grub pests of sugar cane in Somalia.

Le Pelley [6] lists five Dynastinae, four Melolonthinae and three Rutelinae from Kenya, Uganda and Tanzania, which feed upon sugar cane. In Tanzania, *Cochliotis melolonthoides* (Gerstaecker) was studied in some detail by Jepson [7]; more recent studies and reports include those of Bjorking and Spry [8], Waiyaki [9,10], Luhanga [11], Luhanga *et al*. [12], and Saidi *et al*. [13]. There is a detailed report by Sweeney [14] on the sugar cane pest species in Swaziland, updated by Williams [15]. The dynastine *Heteronychus licas* Klug is the most important white grub pest of sugar cane in Swaziland [14,16], parts of South Africa [17] and is a problem as far west as Nigeria [18].

Jepson [7] studied *C. melolonthoides* for two years in Tanzania, at the Arusha Chini Estate. The adults swarm from the first showers of the main rains in early October to the end of November, although there may be a subsidiary swarm at the beginning of the minor rains in March. The eggs which take 15 days to hatch are common in December. First instar larvae feed on humus and organic matter in the soil often at depths of up to 91 cm (36 inches); the second and third instars move up to feed upon roots much nearer the surface. The larvae develop to the third instar by June and continue feeding until August when they descend to a depth of 46–91 cm (18–36 inches) to pupate. The pupa last about 14 weeks before the adults emerge en masse again in October. Flight was recorded as lasting only from 18:40 h to 19:05 h and the beetles sometimes swarm around nearby trees. The adults were not observed to feed, and Jepson suggests the flight is purely for mate location. Jepson used a sample unit of 91 × 61 × 30.5 cm deep (36 × 24 × 12 inches) incorporating a cane stool (a primary shoot and its surrounding tillers) and its root system; it is not clear whether the quadrat was aligned across or along the cane row. Based on his experience in Tanzania and Mauritius he suggests that at 1000–8000 larvae/acre a light infestation is evident, at 8000–20,000/acre a moderate infestation and at 20,000–100,000/acre a heavy infestation; he suggests an economic damage level of 2–2.5 larvae per stool (8000–10,000/acre).

Jepson [7] also observed the natural enemies of *C. melolonthoides* in Tanzania. *Campsomeris mansueta* (Gerstaecker), a large scoliid wasp, parasitized third instar larvae and may have accounted for 20%–50% mortality of that stage. Smaller species of *Campsomeris*, *C. felina* (Saussure), *C. lachesis* (Saussure) and *C. caelebs* (Sichel), attack second instar larvae but account for only 10%–25% mortality of this stage between them. A nematode was found in 2 out of 500 larvae, and considered of no significance at this level. Predatory beetle larvae, and asilid fly larvae were also present and probably capable of killing first instar larvae, but ants were not discussed. The incidence of predation, particularly of a soil living insect, is difficult to assess, but based on his observations, Jepson suggests predation might account for about 50% total larvae mortality.

Subsequently, Hocking [19] reported that a fungus, *Ophiocordyceps barnesii* (=*Cordyceps barnesii*), killed mature larvae of *Cochliotis melolonthoides* at Arusha Chini. Sung *et al.* [20] examined the phylogeny of *Cordyceps* and transferred this and many similar fungi to the genus *Ophiocordyceps*. In studies subsequent to those of Hocking, nearby in north-east Tanzania (Moshi), the only natural enemy Waiyaki [9,10] found was a “*Cordyceps* sp” which was widespread in the estate, especially along the moist irrigation canals. This would also be *O. barnesii*, which is further documented by Evans *et al*. [21]. However, *O. barnesii* was originally described from Sri Lanka and Evans [22] suggests that the African population is unlikely to be conspecific. The fungus infects only third instar larvae and the mummified remains from which the stromata develop are typically found in the top 3 cm of soil [21].

**Figure 1:**
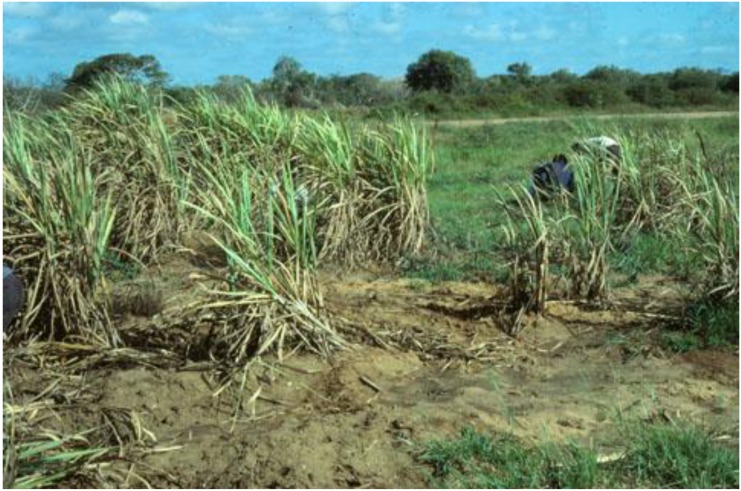
Damage to sugar cane associated with white grubs, Juba Sugar Project, Somalia, October 1986. Note rough grassland in the middle distance, and “bush” in the distance.

At the Juba Sugar Project (JSP), Somalia, a combination of factors contributed to crop loss in the 1980s including poor drainage, soil types, warthogs and wild pigs, goats, cows, weeds, termites, as well as white grubs, although when the JSP was established, stem borers were considered the main insect pests [23,24]. In 1984 small local pockets of white grub infestation started to be noticed. A collection of insects associated with white grub damage was sent to CABI for determination around 1984; it included adults of *Cochliotis melolonthoides*, *Schizonycha* sp. and *Triodontella* sp. Larvae were also sent, but the association of larvae and adults in the case of *Triodontella* sp. and *Schizonycha* sp. was only tentative. Subsequently, surveys were made at JSP and the large numbers of white grub larvae obtained in some areas was cause for alarm. The highest incidence of damage by white grubs was found in localized patches around the edge and in centers of some fields (Figure 1). These surveys were carried out using quadrat samples of 0.5 × 0.5 × 0.5 m placed in the cane row (see Section 2.2).

In May 1986, E. Tremblay and A.N. Jama spent two days at JSP. They collected with lights in the sugar cane and found at least four species of adult Scarabaeidae of which *Schizonycha* sp. was dominant. In their short report, Tremblay and Jama [25] suggest that since irrigation of the cane had been reduced since 1984, this may have caused the white grub problem by negating natural mortality factors such as pathogens. They went on to suggest improved irrigation might resolve the problem. It is against this background that MJWC spent 10 days at JSP at the end of October 1986 (the beginning of the minor rains) looking at the entire white grub complex [26], and GBA spent two weeks at JSP in late April and early May 1987 (the beginning of the main rains) focusing on the two species of greatest concern, *C. melolonthoides* and *Brachylepis werneri* [27].

## 2. Experimental Section

The studies reported here were carried out at the former Juba Sugar Project (JSP), south-western Somalia [23]. The JSP was established and operated by Booker Agriculture International (now part of Booker Tate) to grow sugar for local consumption and export, but was closed down by 1991 with the outbreak of civil war in Somalia. It was located on the flood plain west bank of the Juba River, just south of the town of Jilib, from approximately 0.30N to 0.51N and 42.68E to 42.77E (Annex Figure 3.14 in [28]). The JSP was destroyed in Somalia’s civil war and the land reverted to pastoralists [29]. Examination of the JSP site using Google maps (2012) indicates that most of the area is now grazed, some is cultivated and some is inhabited. Because of these events over the last two decades, it has not been possible to assess changes in the white grub problem, or whether white grubs cause crop losses in the limited arable crops now present in the area.

The JSP soils were categorized as a mixture of fine, medium and coarse alluvial soils, on which the sugar cane was grown under irrigation throughout the year, and an area of marine plain unsuitable for cultivation. At the time of our visits three varieties made up the bulk of the sugar cane planted: Co 997, NCo 376, N53/216. The main rains (locally known as the gu rains) occur from the end of March to mid-July, and the minor rains (locally known as the deyr or der rains) from the end of September to early December, but rainfall is highly variable and periods of drought common.

At JSP, sugar cane was grown in blocks of about 10 hectares [23], and each block was divided into about 12 fields. The northern half of the estate was termed Labadad South (LS) and comprised some 35 blocks, while the southern half was called Kamsuma North (KN) and covered 45 blocks. Abbreviations are used here (as they were at JSP) to designate particular fields, thus, KN06A/01 would be field 1 in block 6A of Kamsuma North *etc*. The Management Compound (MC) was in the center of the estate, and some of the original “bush” (Figure 1) was preserved within this area.

It should be pointed out that even at this time the infrastructure in Somalia was limited and many items taken for granted in most developing countries, e.g., plastic vials for medicines and kitchen containers that can be adapted for insect handling and rearing were simply not available. Hence, some of the materials used were based on what was available, e.g., oil drums and drink cans, rather than what would have been desirable.

### 2.1. The White Grub Species

The taxonomy of beetles (like most insects) is based upon the adult stage. Hence, although it is the larvae of white grubs that cause most or all of the damage, the identification is mostly based on adults. Although larvae of different species are superficially similar, in Africa it has been found that many species can be distinguished by details of the raster (the arrangement of spines and hairs at the posterior end) which incorporates the pallidium (a compact arrangement of spines anterior to the anus), the shape of the anus, and characters of the mouthparts [1,14]. Thus, sampling the cane fields yielded at least seven recognizable morphospecies of white grub larvae based on examination of their rasters, while collecting at light produced rather more adults of genera known to have white grub larvae. Some of these were matched to adults during this study, others remain to be associated. As each morphospecies was recognized it was allocated a code letter: A, B, C, *etc*. for larvae, and Z, Y, X, W, *etc*. for adults. All morphospecies were checked by taxonomists of the CAB International Institute of Entomology at the Natural History Museum, London, the larvae by M.L. Cox and the adults by R. Madge. As a result, one additional uncommon ?*Schizonycha* sp. larval morphospecies was recognized (Section 3.1.7), and several additional adult *Schizonycha* spp. (Section 3.1.16). In preparing this paper, the identities of the two largest species were reassessed based on recent taxonomic reviews (Section 3.1).

At JSP, the known white grub pests are all Melolonthinae, but representatives of Rutelinae and Dynastinae are also common at light and could have larvae attacking sugar cane. For example, *Heteronychus abyssinica* Jack was quite common at lights at JSP, but commoner in the Management Compound than in the cane; the congeneric *H. licas* is a known pest of sugar cane and other crops [30] and reported from sugar cane in Somalia [4]. As all studies were on-going simultaneously, understanding of the species and the correlation between larvae and adults improved over time. Each morphospecies is briefly introduced here to establish the species of concern, although the information presented is derived from the studies and observations that follow. Voucher specimens of all recognized larvae and adults were placed in the Natural History Museum, London.

There are three instars in the development of all white grubs [1]. During each instar the head capsule, which is chitinized and rigid, remains the same size, while the body grows as the initially wrinkled skin is stretched to its maximum expansion. Hence, recognition of the different larval instars was based on head capsule size. After the October 1986 visit, MJWC examined preserved larvae, and measured the width of each head capsule. Based on this measurement, observations made with a hand lens in the field were confirmed and larvae of each species could be divided into instars. Not all instars were present, and where only one or two instars were present the allocation of instar number was provisional (e.g., larvae of *Schizonycha* spp., unidentified Melolonthinae sp. and unidentified Cetoniinae sp.).

If this white grub problem were studied today, DNA barcoding [31] could have been used in combination with traditional taxonomy tools to rapidly match white grub larvae with adults, help clarify any cryptic species, and help identify all stages. Recent work in South Africa has begun to develop this approach [32]. 

### 2.2. Distribution of White Grub Larvae at JSP

Samples were taken from various sugar cane blocks and fields before, during and after the visits of MJWC and GBA, under the direction of JSP and BAI staff. For each field, 20 standard JSP 0.5 × 0.5 × 0.5 m pits were dug. These were arranged along the four equally spaced lateral irrigation lines, five sites per line, paced out at 10, 80, 160, 240 and 310 m from the edge of the field adjacent to the hydrant line. The quadrat was placed in the cane row, four rows across from the lateral line. The objective was to obtain samples from different fields in the blocks affected by white grubs, to assess the spatial and temporal distribution.

Because the diversity of white grub species was not recognized prior to October 1986, all white grubs were pooled prior to this date. However, it seems likely that most of the white grubs counted belonged to the two large, priority species, *C. melolonthoides* in the north of JSP and *B. werneri* LaCroix in the south. For samples taken after October 1986, the two priority species were distinguished together with their three larval instars.

There were other problems with some of the early samples. Pits were of irregular dimension, up to 1 m on one side; a measuring stick was introduced to produce regular quadrats (including depth). Not all pits were being dug in the cane row; some were in the inter-row but this was corrected.

### 2.3. Distribution of White Grub Larvae in the Soil in Relation to the Cane Row

Jepson [7] describes, and illustrates an example of, a method of making transects across the cane furrow to record the distance from the center of the furrow, and depth of all larvae found (in inches). This method was used to (1) obtain comparable data for the situation in Somalia, (2) to assess the effectiveness of the 0.5 × 0.5 × 0.5 m quadrat samples being used in the general survey at JSP (Section 2.2), (3) to check for the presence of pupae of *C. melolonthoides*, and (4) obtain material of early stages of all white grubs for evaluation and identification. Four similar transects were dug in JSP in December 1986: Two in KN06A and two in LS10/09.

A section of furrow of length 91 cm (36 inches) was selected so as to incorporate the center of a stool or group of stools, and the width of the transect to 61 cm (24 inches) on each side, marked out. A pit of 131 cm (51 inches) depth was then excavated in successive layers of 7.5 cm (3 inches). Within each layer, the distance was recorded at which each larva occurred from the center, and the larvae were categorized into instars and types (A, B, C, D *etc*.) based on the form of the raster. Although care was taken not to move the soil as it was searched, there was doubtless some movement of larvae during this process; however, because excavation was carried out in successive layers, the depth records should be accurate.

### 2.4. Sampling Adult Scarabaeidae Attracted to Light

A 45 gallon oil drum was converted to make a mercury vapor light trap similar to the Robinson trap [33], but much deeper. It was operating with a 125 watt mercury vapor bulb and ballast. Trap localities were either in the JSP Management Compound where electricity was available throughout the night, or in the sugar cane blocks, where electricity was provided from a trailer mounted generator. The Management Compound contained stands of original riverine forest and was considered to represent the local natural or “bush” habitat. The trap was emptied by tipping the contents into a plastic box, which was sealed, labeled and placed in a freezer until the contents could be processed. Adult Scarabaeidae were identified to species or genus and counted.

Preliminary observations using the light trap in the JSP Management Compound supported earlier reports [7] that Scarabaeidae beetles fly primarily in the early evening. Hence, for the first visit in December 1986, it was decided that the trap need be run only from dusk until 22:00 h. During three nights in April 1987, this was assessed more rigorously by emptying the trap at four intervals during the night, and recording the numbers of *C. melolonthoides* caught for each time period separately. These numbers are not independent (beetles trapped in the first part of the night might otherwise have continued flying and been caught later) and so are not analysed further.

In October 1986, at the beginning of the minor rains, the light trap was run on four evenings in the cane fields and two nights in the Management Compound. On the second visit in April–May 1987, heavy rain at the belated onset of the main rains meant that access to the sugar cane by vehicle was very difficult, and the trap was mostly operated in the Management Compound. Between our two visits and for several nights after the second visit, the trap was run by JSP staff. The numbers of Scarabaeidae beetles captured were very variable and sometimes large, which meant that on some occasions, recording all white grubs captured was impractical, so counting focused on *C. melolonthoides* and *B*. *werneri*, the two larger, individually more damaging species, which were assumed to be of greater economic importance because of their much lower damage threshold (Section 3.9). Given the apparent changes in flight activity during the night and the diversity of trapping periods during the night (Section 3.4), the results are not analysed or condensed further.

### 2.5. Reproductive Phenology of Cochliotis melolonthoides Based on Light Trap Samples

Adults of *C. melolonthoides* were present from the onset of the main rains in April and May, when large numbers were obtained from the light trap, but not in the minor rains in October. An analysis of the *C. melolonthoides* adults captured April–May 1987 was made. For either the entire sample, or taking a subsample when the number caught was large, each individual was sexed, and females were dissected to assess whether they were gravid or not, and on some occasions the number of mature eggs was counted.

### 2.6. Reproductive Phenology of Cochliotis melolonthoides and Brachylepis werneri Based on Field Samples

Irregular pit samples were dug in April and May 1987 to look for pathogens of white grubs. The number and stage of the two principal species were also recorded. Sample sites were selected under badly damaged cane and the fields sampled had previous records of high infestation. Soils were mainly sandy loams and bare patches were evident (Figure 1).

### 2.7. Rearing Field Collected Larvae

At the beginning of this study, only one larva had been associated with its adult and named: *C. melolonthoides*. Hence, in parallel with other studies, an attempt was made to rear through field collected larvae of types B–F in order to link them to the corresponding adults, which could be identified. The following approach was used as most likely to produce results with minimal technical supervision between our visits.

Four 45 gallon oil drums were cut in half to give eight cylindrical half drums. Small holes were made in the base for drainage. Once the inside of the drum had been thoroughly cleaned, the base was covered inside with insect mesh and drums were filled with soil and a stool of sugar cane planted in each. In this process, soil and stools were taken from a field where no white grubs had been found in surveys, and the soil was first sifted by hand to check for the presence of white grubs. It was then necessary to cover the tubs with the cane stools planted so that the tub was ventilated and light, but no access or egress for Scarabaeidae adults was possible. Mosquito nets were cut up and made into cones; the base of the cone was then tied tightly around the top of the drum and the top tied to a prepared framework. The drums were placed against a south-east facing wall so that they received direct light for the first part of the morning only. The half-drums were set up on 1 November 1986 as shown in Table 1.

**Table 1:** Rearing trials studies set up in half-drums, 1 November 1986.

Drum	Larva species andsource	Number and stage
1,2	*Brachylepis werneri* (larva F) from LS10	10 instar III larvae
3	Unknown Melolonthinae sp. (larva D) from KN13	5 instar II and III larvae
6	?*Schizonycha* sp. (larva C) from KN06A, KN13 and LS10	6 instar III larvae
4,5	*Anomala ancilla* (adult W) from light trap	5 adult females with developed ovaries
7	?*Triodontella* sp. A (larva B) from KN06A and KN13	26 instars II and III larvae
8	?*Triodontella* sp. A (larva B) from KN06A and KN13	28 instars II and III larvae

The larvae used for these field trials were taken from field samples, and although the larvae used appeared healthy, it is almost inevitable that, in spite of the care taken, the process of excavation and transport to the laboratory would have caused damage and trauma, leading to early death of some larvae.

The maintenance procedures anticipated included the following. The temperature in the drums was to be monitored and if high (>25 °C around cane roots) additional shading would be provided. The set up with these nets was somewhat insecure and if found to be adversely affected by high winds, a more secure arrangement would need to be devised. The drums were to be watered two times a week, by using a hose which can add water through the mesh tent, so that it need not be opened. At the same time a check should be made for any adult beetles. Any found should be removed (except for the adults already present in drums 4 and 5), preserved and labeled as to which drum they emerged from and when. Emergence of adults was considered most likely to be at the beginning of the next main rains in May. BAI/JSP staff excavated Drums 1, 4 and 8 to check on progress in November 1986, and the remainder was examined by GBA at the beginning of the main rains at the end of April.

Disappointing results were obtained using the half drums (see Section 3.7), so a small scale rearing method was tested and used for bioassays. Field collected eggs of *C. melolonthoides* were set up in individual containers (drink cans with the tops removed). The eggs were placed in soil and covered with moistened tissue. A rearing system for ?*Triodontella* sp. A (larva B), unknown Melolonthinae sp. (larva D) and unknown Cetoniinae sp. (larva E) in similar plastic containers containing soil and grass were not successful, due to an abundance of nuisance ants, *Paratrechina* sp. which adversely affected the larvae.

### 2.8. Alternative Hosts for Larvae

White grubs are usually considered polyphagous, at least amongst Poaceae. Local crops such as maize, millet and sorghum may be vulnerable, and wild grasses are likely hosts. In a clearing in LS10/11 (Figure 1), three quadrats were dug amongst grasses and compared with quadrats from the adjacent cane row. The quadrats in the former area were selected by eye as representative, while those in the cane row were selected at random from within the line of the cane row.

### 2.9. Extrapolating Population Density—Damage Relationships

It was apparent from the samples taken by JSP staff, and the observations reported above that the distribution of white grubs within a field is highly contagious. Large differences can occur between apparently similar sites very close to each other, e.g., in Section 3.3, pits 3 and 4 were excavated under almost identical stools yet one had more than twice as many white grubs as the other. Similarly, the population of white grubs may not be closely linked to the apparent damage, e.g., in Section 3.3, pits 1 and 2 represent heavily damaged and relatively healthy stools a few yards apart, yet sample 2 actually has more large white grubs (instar 3 *C. melolonthoides*) than sample 1.

Clearly any attempt to link white grub populations with the damage caused will have to be based upon extensive studies. Any economic threshold value will be dependent upon the age of the crop, soil types and water stress as well as white grub species, stage and season. An alternative strategy is to extrapolate from published findings for the same or other white grubs attacking sugar cane elsewhere. Thus, Jepson's [7] figure of 2–2.5 larvae per stool (or 8000–10,000/acre = 20,000–25,000/ha) for *C. melolonthoides* in Tanzania provides a standard. An approach was developed to estimate how this threshold might need to be modified to allow for the body size of the larvae of different white grub species. This was based on the assumptions that the amount of damage each larva does is a function of its body mass, and that body mass is a linear function of body volume. The former is a simplification of a complex interaction between white grubs and the sugar cane, but the later has been subsequently demonstrated, at least for Neotropical white grubs [34].

During the October 1986 visit, field collected white grubs were preserved by immersing them in boiling water for 30 seconds for small larvae or up to two minutes for large larvae, and then placing them in 70% ethanol. A total of 70 larvae of six different species were thus preserved.

For each, the head capsule width was measured using a binocular microscope and a calibrated eye-piece graticule. The volume was measured by noting the change in level when a larva having been dried off with tissue paper was immersed in a partially filled measuring cylinder. In the case of the smallest larvae, they were measured in batches and the volume averaged. The bodies of the preserved larvae were more or less fully distended, due to the preservation method, so the volume is likely to be comparable to the final instar size. Larvae that were damaged or distorted were not included in the volume measurements.

The resultant volumes were plotted against head capsule width, and two models fitted to the relationship by linear least squares regression analysis (using the data analysis add-in in Excel): One based on a log-linear relationship, and the other on a cubed power. The equations were then used to calculate the economic threshold equivalence for third instar mature larvae of the different species. Since the results are compared with the results obtained in the same way for third instar *C. melolonthoides*, the approach should be valid.

### 2.10. Field Observations on Population Density—Damage Relationships

As noted in the introduction to Section 2.9, the distribution of white grub larvae is very patchy, and the correlation between larval density and visible damage was not obvious in casual observations. To test this more objectively a scoring method for damage was prepared, so that larval densities in soil samples could be taken from stools different categories of damage.

In consultation with Mr. Peter Drew, BAI agronomist, a damage index was drawn up as follows:

1 = all tillers are healthy;
2 = up to 1/4 of tillers are yellow and stunted;
3 = 1/4 to all tillers are yellow and stunted;
4 = all of stool stunted, some dead or dying tillers; and
5 = stools either completely dead or only a few green tillers which are small and unhealthy.

In November 1986, three fields in block KN13 were selected by Mr. Drew, where all levels of damage index were present. Under his supervision, two sets of twenty standard pits of 0.5 × 0.5 × 0.5 m were subjectively located under stools in each damage category, and the number of instar 3 *C. melolonthoides* counted in each. The numbers were pooled for each set of 20 samples, giving 30 data points. Of the three fields sampled, KN13/08 was var. NS3-216 and the other two were C0997. The soils of these fields were fine grain with a very high percentage of silts, and the block was previously cut on 3 August 1986. The mean of each set of 20 quadrats was used to calculate a linear regression against damage index using the data analysis add-in in Excel.

### 2.11. Natural Enemies of White Grubs

The action of natural enemies can provide the foundation of an integrated pest management approach to pest control, while individual components of the natural enemy complex can be manipulated to augment their impact. Accordingly, evidence for the presence of natural enemies of white grubs was collected at JSP.

#### 2.11.1. Field Observations

During field work at JSP, observations were made to detect predators, parasitoids or pathogens acting on white grubs. Common predators would have been observed in the course of other work; if these were predators of white grubs, predation events might be observed, such as ants actively feeding on larvae. Larval remains that may have been killed by predators would also have been found, especially when sieving the soil in quadrat sampling. To detect parasitoids, larvae were checked for evidence of ectoparasitism, the soil from the transect samples was sieved for pupating parasitoids, and samples of white grub larvae were dissected in the laboratory for evidence of internal parasitism. In addition, the dissection of adults to assess reproductive state (above) would have revealed well developed larvae of parasitoids of adults. Pathogens would have been detected as cadavers killed by bacteria or viruses, or mummified corpses with fungal fruiting bodies, which in the case of *Ophiocordyceps* spp. would be rather conspicuous.

#### 2.11.2. Survey for *Ophiocordyceps barnesii*

In field observations and samples, *Ophiocordyceps barnesii* was found infecting third instar *B*. *werneri* in Labadad South only. Further observations were made by digging sample holes in the fields known to be infected with this pathogen (LS10/09 and LS10/08). Samples were not standardized, extending as appropriate to include the whole root system or until no material was being found, but the size of each hole was recorded. The fields sampled were bare and patchy in places, and densely overgrown with sorghum grass.

#### 2.11.3. Sampling for entomopathogenic nematodes (EPNs)

Soil from block KN13/08 collected on 27 April 1987 was placed in individual drink cans and one first instar larva of *C. melolonthoides* was added to each container. At this time only first instar larvae were available, otherwise third instar larvae would have been used. After one week, on 4 May 1987, the larvae were checked for evidence of infection by EPNs, although if time had not been limiting this would have been done after ten days. In the one case where this was found, five third instar *B*. *werneri* larvae were added to the soil sample, and checked for evidence of EPNs after three days.

## 3. Results

### 3.1. The White Grub Species

Adult beetles were not observed feeding on sugar cane above ground at JSP, so unless adults emerge from the pupa with ovaries developed (which does not seem to be the case), adults must feed to develop eggs in the ovaries before oviposition takes place. The lack of adult feeding on sugar cane above ground implies that either females feed below ground, or they feed on plants other than sugar cane. These possibilities were not investigated.

#### 3.1.1. *Cochliotis melolonthoides* (Gerstaecker) (Larva A)

**Figure 2:**
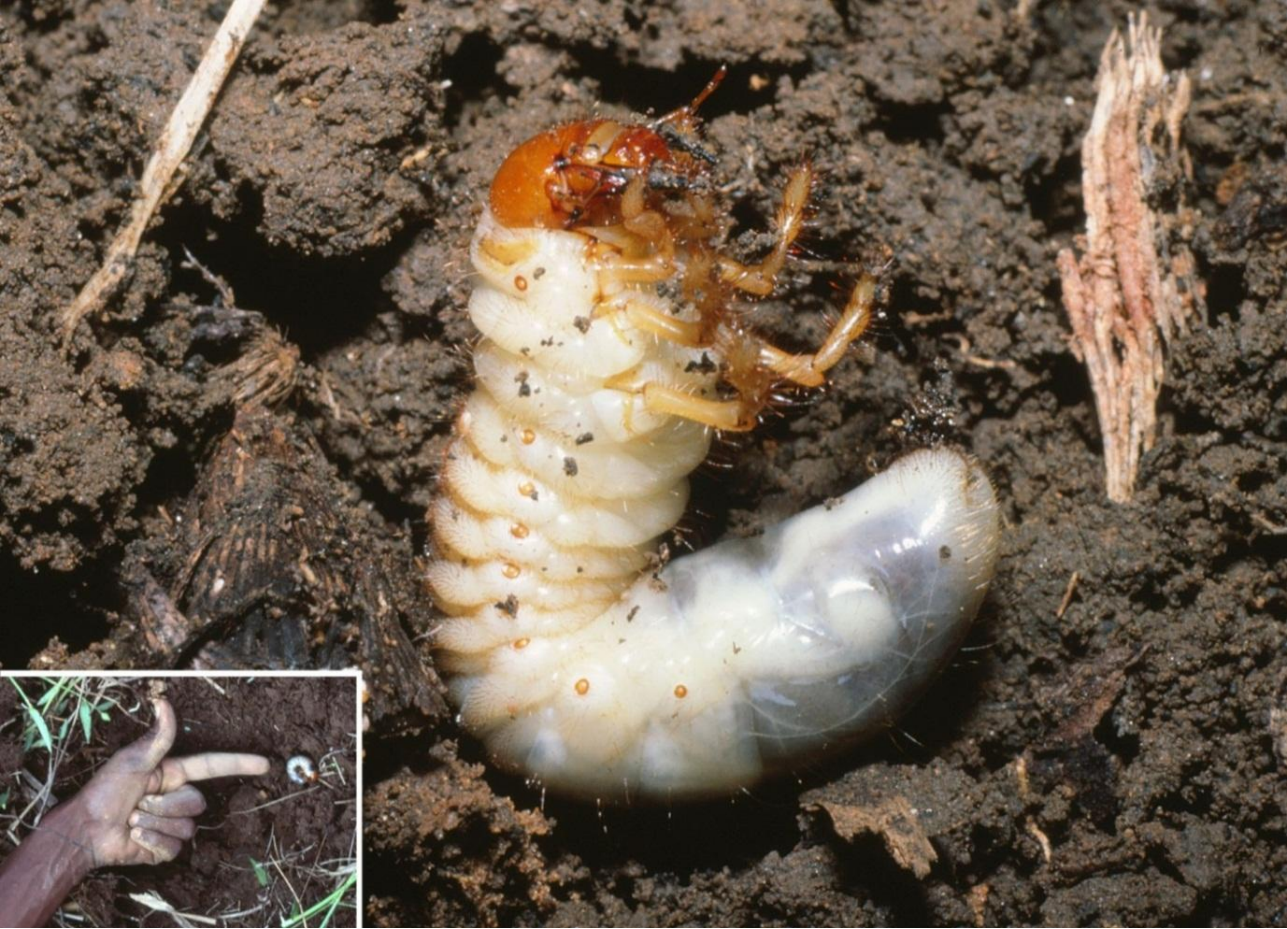
Third instar larva of *Cochliotis melolonthoides*, Juba Sugar Project, Somalia, October 1986.

**Figure 3:**
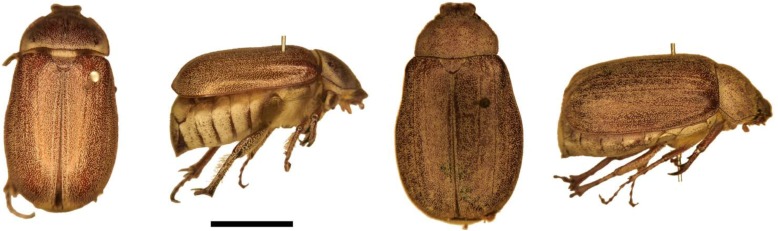
Adults of the two most individually damaging white grubs at the Juba Sugar Project, Somalia. **Left**, *Cochliotis melolonthoides*, and **right**, *Brachylepis werneri*.

Lacroix [35] revised the East African genus *Cochliotis* and considered that only the newly described *C. somaliensis* Lacroix occurred in Somalia. Based on Lacroix’s treatment, we confirmed that voucher material of the species we found as a sugar cane pest at JSP is *C. melolonthoides*. However, it is not impossible that both species were mixed together under this name in our samples of adults. During the October 1986 visit, larvae of this species were found in samples in KN06A and KN13, but not in LS10. Almost all larvae were third instar (Figure 2), but a few were second instar; no pupae were found, and light trapping produced no adults. Adults (Figure 3) were light-trapped in large numbers during the main rains at the second visit in May 1987. Hence the life cycle is probably as follows: The adults appear in a mass emergence at the beginning of the main rains, they develop eggs and oviposit. The larvae probably complete feeding around the end of the minor rains or soon after, before descending to at least 61 cm (24 inches) depth to pupate. Adult emergence from the pupa is probably just before the main rains, but if so the adults remain in the soil until the rains start.

**Figure 4:**
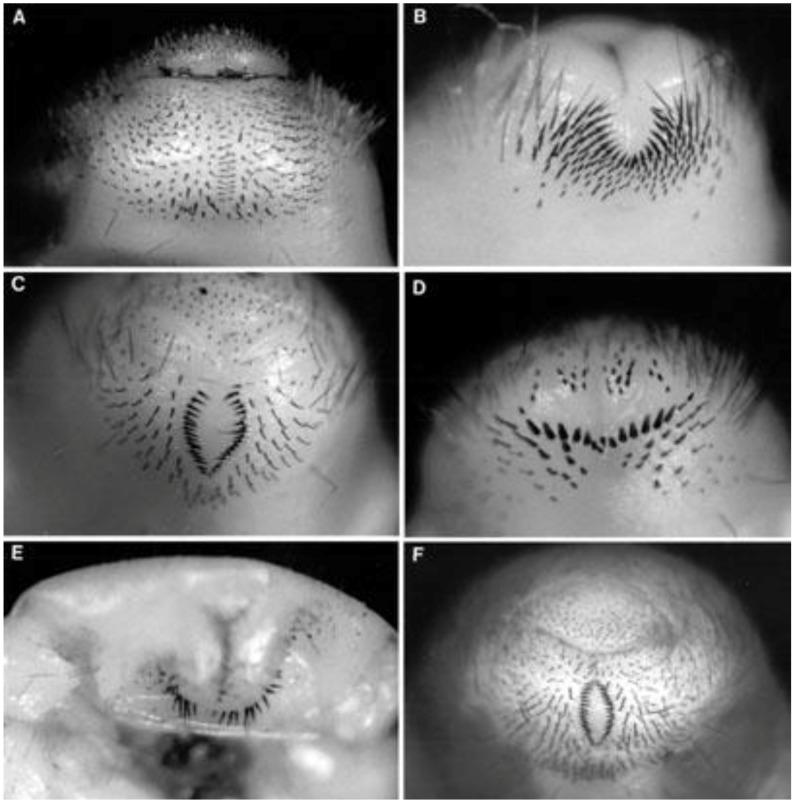
Rasters of white grubs found at JSP. A, *Cochliotis melolonthoides*; B, ?*Triodontella* sp. A; C, ?*Schizonycha* sp.; D, unknown Melolonthinae sp.; E, unknown Cetoniinae; F, *Brachylepis werneri* (the letters correspond to the code letters used). Photographs by A. López-Ávila.

The larva has a distinctive raster (Figure 4A; [7]) notably the pallidium is inconspicuous to the naked eye, but consists of a double parallel row of inward directed spines which overlap, or nearly do so, while the anus is arc shaped, not y shaped. The raster of *C. somaliensis* has not been reported [35], so although we saw no significant variation in material from JSP, the possibility that both species of *Cochliotis* were present cannot be assessed until details of the raster of *C. somaliensis* are known. In October 1986 this was the most important white grub present at JSP; it was associated with economic damage in blocks KN06A and KN13.

#### 3.1.2. ?*Triodontella* sp. A (Larva B)

In October 1986, larvae of this type were common in KN06A and KN 13, but almost absent in LS10. This larva belongs in the tribe Sericini which includes the genus *Triodontella*. Given the distribution of larvae, it is likely that it is the larva of the adult identified as *Triodontella* sp. A, which was very common at light in KN06A but very rare in LS10. First, second and third instar larvae were found but firsts were rare. If the larva and adult *Triodontella* sp. A are correctly associated, this species is breeding more or less continuously in the sugar cane.

Superficially this larva is most distinctive due to silvery white fat bodies (*vs*. yellowish white) visible through the cuticle and extending into the anal region. The raster (Figure 4B) is distinctive; with the naked eye; a dark U of spines can be seen before the anus which is clearly Y shaped.

#### 3.1.3. ?*Schizonycha* sp. (Larva C)

Larvae of this type were found in KN06A and LS10. They were probably instar III and not common. In a rearing trial reported below one adult *Schizonycha* sp. probably *vastatrix* Chiaromonte (species X) was reared from larvae of this form, but this single result needs confirmation. The raster (Figure 4C) has the palladium consisting of two adjacent arcs (similar to larvae F and G). The arcs meet in a pointed tip anteriorly but are divergent posteriorly. The anus is Y shaped, but the tail of the Y is short and potentially overlooked. From our observations, this species was not associated with economic damage; but Tremblay and Jama [25] found one (or more) *Schizonycha* spp. dominant at light in KN06A in the main rains in May 1986, so the situation is probably changeable.

#### 3.1.4. Unknown Melolonthinae sp. (Larva D)

Larvae of all three instars were found in KN06A and KN13 in October 1986. Larvae had also been collected at JSP in May 1986 and sent to the CAB International Institute of Entomology for identification, suggesting two generations a year or continuous breeding. In the raster (Figure 4D), the transverse row of spines in front of the Y shaped anus is distinctive. Because these spines are directed straight out from the body, they are actually longer than they appear to be in the figure. The white fat bodies of the living larva extend into the anal region. This species appears to match Sweeney's Melolonthidae species Q [14]. At the numbers found, this larva is of no economic significance.

#### 3.1.5. Unknown Cetoniinae sp. (Larva E)

A few larva of instar II or III were found at LS10 in October 1986. They were all found below 46 cm (18 inches) depth, and like other Cetoniinae are probably not root feeders. The body is a yellowish white due to the fat bodies, and a darker dorsal line is usually evident due to a gap in the fat bodies. The anal region lacks fat bodies and is brownish. The raster (Figure 4E) has two rows of spines in front of the y shaped anus; the two lines are slightly angled to each other and there is a gap between them. Since it is unlikely to feed upon roots, and was scarce anyway, this species was not considered an economic pest.

#### 3.1.6. *Brachylepis werneri* LaCroix (Larva F)

Adults of this species were originally identified as “*Brachylepis* ?*elephas* (Gerstaecker)” by R. Madge and reported as such by Allard [27]. Re-examination in light of the recent generic review by LaCroix [36], showed that they match *B. werneri*, which LaCroix described from southern Ethiopia. Third instar larvae of this species found in LS10 in October 1986 were initially confused in the field with those of *C. melolonthoides* which they resemble in size, although the rasters are distinct (Figure 4). All larvae of this species found in the transects in LS10 were found in the top 15 cm of soil. This suggests they may be particularly susceptible to natural enemies compared to *C. melolonthoides* which is also found much deeper.

The pallidium of the raster is very distinct (Figure 4F), consisting of two arcs of inward facing spines whose ends meet but are bowed in between. Otherwise this larva resembles that of *C. melolonthoides*, including the arc shaped anus. Younger instars may prove to be confusingly similar to the larvae of *Schizonycha* spp. (larvae C and G), but these have not been recognized. This species would seem to be the one which occurred in outbreaks in blocks LS16 and LS10, but in 1986–1987 it was only moderately common in LS10. Adults were only caught at light in June 1987.

#### 3.1.7. ?*Schizonycha* sp. (Larva G)

This species was not distinguished from ?*Schizonycha* sp. (larva C) during our visits. It was subsequently recognized from amongst preserved material from KN06 and LS10. Superficially this species resembles larva C, but the pallidium is more symmetrical, having comparable gaps between the arcs of spines anteriorly and posteriorly.

#### 3.1.8. *Triodontella* sp. A. (Adult Z)

The small adults of this species were found rarely while digging under cane stools in KN06A and KN13 in October 1986. Much larger numbers were obtained by use of the light trap in KN06A (e.g., 1,023 on 25 October); in comparison small numbers were taken in the JSP management compound, KN03, KN13 and LS10 (Section 3.4). There were no specimens amongst the material collected at light in May 1986, but this species could have been ignored due to its small size. It was, however one of the species from soil samples in May 1986 submitted for identification.

In the males, one of the claws of the fore leg is modified into an enlarged, flattened shape while in the female they are normal; this difference can be seen with a hand lens. In the light trap sample from KN06A sexes were present in equal numbers. Dissection of females in October 1986 at the beginning of the minor rains showed no development of ovaries.

Amongst the series of adults taken at light at KN06A were a few small specimens with slightly different scaling which may represent an additional species; these need further study.

#### 3.1.9. *Triodontella* sp. B (Adult Y)

This species was taken rarely and may not be associated with sugar cane. It is browner and smaller than *Triodontella* sp. A and the underside of the abdomen is relatively free of scales.

#### 3.1.10. *Schizonycha* sp. probably *vastatrix* Chiaromonte (Adult X)

In October 1986, this species was quite common in the light trap in LS10 (34 males, 20 females on 28 October) but rare elsewhere (e.g., at lights in the management compound). The fore leg tarsi of the males are much longer in males than in females. Dissected females from this material showed no development of the ovaries.

#### 3.1.11. *Anomala ancilla* Gerstaecker (Adult W)

Adults were moderately common in the light trap in all areas in October 1986 (Section 3.4). The sample of 28 from LS10 was examined more closely: It consisted of 3 males, 25 females and the females had developed ovaries containing white oval eggs 2 mm × 1.3 mm. The male, which is on average slightly smaller than the female, has the inner claw of the fore leg single, whereas in the female it is split at the tip; with experience this is apparent with the naked eye and can be checked using a hand lens. It was noted that when alive this species is a brownish white in color, but that dried specimen turn brown. Specimens were also captured at light in May 1986 at the beginning of the main rains. Further investigation would be necessary to determine if this species is a sugar cane feeder and what its life history is. At the observed incidence, this species is not an economic problem.

#### 3.1.12. *Adoretus* spp. (Adult V)

*Adoretus somalinus* Benderitter and the slightly larger *A*. sp. near *exsecatus* Machatschke were identified. In October 1986, *A. somalinus* was commoner at the light trap in the management compound than in the cane fields (Section 3.4), and hence, it may not be a cane feeder.

#### 3.1.13. *Apogonia* sp. (Adult U)

At the numbers obtained (maximum 57 in LS10) it is of no importance in the cane, and the larva may well feed on other hosts.

#### 3.1.14. *Schizonycha methneri* Kolbe (Adult T)

No adults were captured in the light trap in October 1986, but it is apparent from preserved material that it was common in May 1986. It seems likely that it is based upon this species that Tremblay and Jama [25] stated that *Schizonycha* sp. was dominant. If this is so, the subsequent scarcity of *Schizonycha* spp. larvae is confusing.

#### 3.1.15. *Coelogenia* sp. (Adult S)

Amongst the material preserved from collections at light in May 1986 were three males and four females. The males have long fore tarsi covered on the inner surface with dense fine hairs. This species is unlikely to be causing economic damage.

#### 3.1.16. Other *Schizonycha* spp.

In addition to the two common species of *Schizonycha* treated above (*S. methneri* and *S*. ?*vastatrix*), several other species of this genus are present at JSP. Of these the most important is *S*. sp. nr. *angulata*: One specimen was preserved from those captured at light in May 1986, occasional specimens were captured in October 1986 at light in KN03, KN13, LS10 and the management compound, while two were dug from the soil under sugar cane stools in LS10. Two other species were represented in the May 1986 collections and three more in collections in the October 1986 collections; none of these could be named.

### 3.2. Distribution of White Grub Larvae at JSP

Around 150 field samples were made between August 1984 and October 1986. Because of the problems alluded to in Section 2.2, these are not presented here, but are summarized as follows for what are assumed to be *C. melolonthoides* larvae. In KN13, white grub were consistently common to abundant in field KN13/01 in all samples in February and November 1985, all months from August 1986 to January 1987, but then scarce in March 1987. White grubs were common in field KN13/02 in February and November 1985, and November and December 1986, but at relatively low numbers in August 1986 and January 1987. In field KN13/08 white grubs were common in November and December 1986 but moderate to low in January, March and April 1987. The other five fields sampled irregularly in this block had moderate to low numbers.

For samples from October 1986 onwards, the numbers of individuals of the three different larval instars of the two prioritized white grub species, *C. melolonthoides* and *B. werneri*, are presented in Table 2. *Cochliotis melolonthoides* was restricted to the southern KN blocks, and *B*. *werneri* to the northern LS blocks. *Cochliotis melolonthoides* was considerably more common (50 per 20 quadrats) than *B. werneri* (6.7 per 20 quadrats). Pupae were not found and the very few adults found are not recorded here.

**Table 2:** Collated totals of twenty 0.5 × 0.5 × 0.5 m pit samples of larvae of *Cochliotis melolonthoides* and *Brachylepis werneri* from different fields in selected blocks at JSP.

	Nov 1986			Dec 1986			Jan 1987			Feb 1987			Mar 1987			Apr 1987			
Block and field number	L_1_	L_2_	L_3_	L_1_	L_2_	L_3_	L_1_	L_2_	L_3_	L_1_	L_2_	L_3_	L_1_	L_2_	L_3_	L_1_	L_2_	L_3_	Mean
*Cochliotis melolonthoides*																			
KN13/01	0	0	180	0	0	248	0	0	52				0	1	0				120.3
KN13/02	0	0	55	0	0	36	0	0	2										31.0
KN13/03				0	0	4													4.0
KN13/05										0	0	0							0.0
KN13/06										0	0	0							0.0
KN13/07	0	0	3																3.0
KN13/08	0	0	69	0	0	173	0	0	4							0	0	0	61.5
KN6A/01	2	1	35	0	0	212	0	0	46	0	0	0							74.0
KN6A/02	0	0	4	0	0	7	0	0	0	0	0	0							2.8
KN6A/09	1	0	13	0	0	5	0	0	1	0	0	2							5.5
KN6A/l0	0	0	39	0	0	282	0	0	72	0	0	0							98.3
Mean	0.4	0.1	49.8	0.0	0.0	120.9	0.0	0.0	25.3	0.0	0.0	0.3	0.0	1.0	0.0	0.0	0.0	0.0	50.0
*Brachylepis* *werneri*																			
LSl0/01	0	0	5							0	0	3				0	1	5	4.7
LSl0/02	0	0	4							0	0	1				0	0	2	2.3
LSl0/03	0	0	9													0	0	1	5.0
LSl0/04	0	0	2							0	0	4							3.0
LSl0/05	0	0	4							0	0	4							4.0
LSl0/06	0	0	5							0	0	4				0	0	0	3.0
LSl0/07	0	0	14							0	0	7							10.5
LSl0/08	0	0	7							0	0	8				0	0	0	5.0
LSl0/09	0	0	4							0	0	8				0	0	3	5.0
LSl0/l0	0	0	53							0	0	10				0	5	2	23.3
LSl0/11	0	0	19							0	0	10							14.5
LSl0/12	0	0	5							0	0	9							7.0
LS16/0l	0	0	2													0	0	0	1.0
LS16/02	0	0	9													0	0	0	4.5
Mean	0.0	0.0	10.1							0.0	0.0	6.2				0.0	0.7	1.4	6.7

A small number of first and second instars of *C. melolonthoides* were found in November only, but most were already third instar. Third instar larvae were patchily abundant in November and December 1986, and still common in January 1987, but in February, March and April there were no larvae of this species. In contrast, small larvae of *B. werneri* were only recorded in April 1987, while third instar larvae were progressively less common in November, February and April.

### 3.3. Distribution of White Grub Larvae in the Soil in Relation to the Cane Row

**Figure 5:**
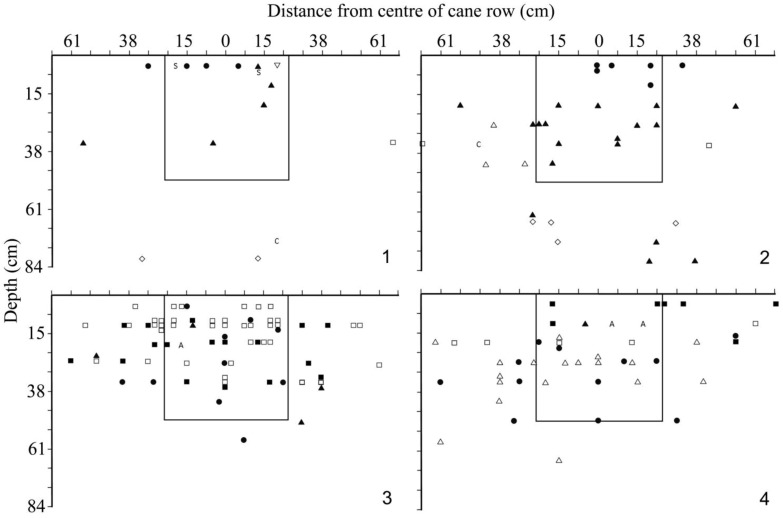
Transects of the cane row to show the distribution of white grub larvae and adults in the soil, JSP, October 1986. The cross section sampled with the normal 0.5 × 0.5 × 0.5 cm pit is indicated by the square. **1**, **2**, KN06/10; **3**, **4**, LS10/09. Key: ● *Cochliotis melolonthoides* L3 (Figure 5.1, Figure 5.2) or *Brachylepis werneri* (Figure 5.3, Figure 5.4); ■, □ ?*Triodontella* sp. A (larva B), L3, L2, L1; ▲, △ ?*Schizonycha* sp. (larva C) ?L3, ?L2; ◊, unknown Cetoniinae sp. (larva E) ?L2; **A**, adult *Triodontella* sp.; **S** *S*. sp. nr. *angulata*; **C**, parasitoid cocoon.

The numbers of each type of larva found are tabulated in Table 3, and the distribution of larvae in transect across the sugar cane row is shown in Figure 5. At this time, only the larvae of *C. melolonthoides* could be positively identified to species, and only third instar larvae of this species were present; all other larvae were categorized as morphospecies.

**Table 3:** The instars and numbers of larvae of white grubs found in four Jepson [7] transects, October 1986.

Species and larval (L) instar		KN06A(1)	KN06A(2)	LS10/09	LS10/09
*Cochliotis melolonthoides*, L3		10	12	0	0
?*Triodontella* sp. A L3		16	8		
	L2	36	5		
	L1	11	0		
?*Schizonycha* sp. (larva C), L?		1	17		
	L3			5	19
	L2				1
Unknown Melolonthinae sp. (larva D) L?		3		1	2
Unknown Cetoniinae sp. (larva E) L?				2	4
*Brachylepis werneri* L3				4	6

Although all transects were excavated to a depth of 130 cm (51 inches), no larvae were found below 84 cm (33 inches), and most (85%) were within 38 cm (15 inches) of the soil surface. In KN06, third instar larvae of *C. melolonthoides* were found down to 84 cm (33 inches) depth, whereas in LS10, the *B*. *werneri* larvae were not found below 15 cm (6 inches).

### 3.4. Sampling Adult Scarabaeidae Attracted to Light

The results from May 1987, emptying the trap at intervals during three nights and recording the number of *C. melolonthoides* for each period are presented in Table 4; 62%–78% were caught during the first part of the night but beetles continued to fly and be trapped throughout the night.

The numbers of each genus trapped each night are summarized in Table 5. Light-trapping from the beginning of the minor rains (October 1986) until the main rains (April–May 1987) showed that almost no adults flew in the dry period between the two rains. It seems likely that none flew in the dry period between the main rains and minor rains, but we have no observations to support this except casual observations by JSP staff that no adult Scarabaeidae were seen at domestic or public lights during this time. Adult *C. melolonthoides* and *B*. *werneri* flew at the beginning of the main rains only—not at the beginning of the minor rains. All the other species flew at the beginning of both rainy periods, although most were in larger numbers at the beginning of the main rains (Table 5).

**Table 4:** Light trap catches of *Cochliotis melolonthoides* over time during the night: Absolute numbers and as a percentage of the total catch of that species for the night, May 1987.

	2 May			10 May			15 May	
Time	Number caught	%	Time	Number caught	%	Time	Number caught	%
18:20–20:20 h	705	78.3	18:30–19:30	638	73.0	18:30–19:30	578	62.1
20:35–21:35 h	100	11.1	19:35–20:30	58	6.6	19:35–20:30	153	16.4
21:40–22:40 h	51	5.7	20:35–21:30	38	4.4	20:35–21:30	102	10.9
22:45– 6:30 h	44	4.9	21:35–dawn	140	16.0	21:35–dawn	98	10.5
Total	900			874			931	

**Table 5:** Adult Scarabaeidae catches in the light trap, October 1986 to May 1987. Key to adults: AD *Adoretus* spp., AN *Anomala* spp., AP *Apogonia* spp., BR *Brachylepis werneri*, CO *Cochliotis melolonthoides*, SC *Schizonycha* spp., TRI *Triodontella* spp., TRO *Trochala* spp.

Location	Date	Time	*AD*	*AN*	*AP*	*BR*	*CO*	*SC*	*TRI*	*TRO*	Total
KN6A	25 Oct 1986	Dusk–22:00h	16	17	0	0	0	5	1023	0	1061
MC	27 Oct 1986	Dusk–dawn	29	93	8	0	0	0	19	0	149
LS10	28Oct 1986	Dusk–22:00h	2	28	57	0	0	54	10	0	151
KN3	29 Oct 1986	Dusk–22:00h	1	1	0	0	0	6	3	0	11
KN13	30 Oct 1986	Dusk–22:00h	1	1	2	0	0	2	12	0	18
MC	31 Oct 1986	Dusk–dawn	1	4	0	0	0	1	3	0	9
KN6A	2 Nov 1986	Dusk–22:00h	0	0	0	0	0	0	2	0	2
KN13	22 Nov 1986	Dusk–22:00h	0	0	0	0	0	1	2	0	3
KN6A	22 Nov 1986	Dusk–22:00h?	4	2	0	0	0	0	6	0	12
LS10	23 Nov 1986	Dusk–22:00h?	0	1	0	0	0	0	0	0	1
LS10	24 Nov 1986	Dusk–22:00h?	0	0	0	0	0	0	0	0	0
KN3	16 Feb 1986	Dusk–22:00h?	0	0	0	0	0	0	0	0	0
KN13	17 Feb 1987	Dusk–22:00h?	0	0	0	0	0	0	0	0	0
MC	29 Apr 1987	18:30–21:30h	0	0	0	0	32	0	3	0	35
MC	1 May 1987	18:30–23:00h	0	0	0	0	707	0	0	0	707
MC	2 May 1987	18:20–06:30h	11	2	0	0	900	6	0	3	922
KN13	3 May 1987	17:45–21:30h	0	19	0	0	144	2	0	0	165
KN13	4 May 1987	17:45–21:30h	0	26	0	0	637	4	70	0	737
MC	5 May 1987	18:20–06:30h	-	-	-	-	98	-	-	-	98^a^
MC	6 May 1987	18:30–22:30h	0	0	0	0	4977	0	0	0	5278^b^
MC	10 May 1987	18:30h–dawn	300	211	2306	22	874	1816	0	0	5529
MC	15 May 1987	18:30h–dawn	0	35	11	0	1031	102	0	0	1179
KN3	17 May 1987	18:15–21:30h	0	61	0	0	1007	341	0	0	1409
KN13	18 May 1987	18:15–21:30h	23	45	113	0	825	416	92	0	1514
LS10	21 May 1987	18:15–21:30h	5	15	5	0	1	11	0	0	37
MC	22 May 1987	18:30h–dawn	38	52	48	0	310	148	0	0	596
LS10	23 May 1987	18:15–21:30h	174	36	274	0	151	99	68	0	802
KN13	24 May 1987	18:15–21:30h	36	14	11	0	175	63	56	0	355
Total			641	663	2835	22	11869	3077	1369	3	20,479

^a ^Only *C. melolonthoides* was counted this night; ^b ^Including 301 white grub beetles of species other than *C. melolonthoides.*

### 3.5. Reproductive Phenology of Cochliotis melolonthoides Based on Light Trap Samples

Adult *C. melolonthoides* were assessed over a month from the onset of the main rains. The data on reproductive status of light-trap caught adults are presented in Table 6. The percentage of males attracted to the light trap varied from 3% to 56% on different nights, with no apparent trends; pooling all material, the average was 18%. Although females were more common than males, for the first two weeks almost none (3%) contained eggs, but in the second two weeks about half (45%) contained at least some eggs.

**Table 6:** Reproductive status of adult *Cochliotis melolonthoides* attracted to light-trap, April–May 1987.

Location	Date^a^	Total *C. melo-lonthoides*	Sample	Males	Females	% male	Immature females	Gravid females	% gravid
MC	29 Apr	32	32	4	28	13%	28	0	0%
MC	1 May	703	703	22	681	3%	657	24	4%
KN13	3 May	144	144	38	106	26%	102	4	4%
KN13	4 May	637	637	83	554	13%	552	2	0%
MC	5 May	98	98	8	90	8%	90	0	0%
MC	10 May	874	433	244	189	56%	169	20	11%
MC	15 May	1031	675	204	471	30%	249	222	47%
KN3	17 May	1007	382	30	352	8%	197	155	44%
KN13	18 May	825	401	32	369	8%	210	159	43%
MC	22 May	310	310	58	252	19%	145	107	42%
KN3	23 May	151	151	22	129	15%	59	70	54%
KN13	24 May	175	175	11	164	6%	89	75	46%
Totals		5987	4141	756	3385	18%	2547	838	25%

^a^ For time of trap operation, see Table 5.

### 3.6. Reproductive Phenology of Cochliotis melolonthoides and Brachylepis werneri Based on Field Samples

The results are summarized in Table 7. Adult *C. melolonthoides* and *B*. *werneri* were found usually just below the soil surface, and eggs were recovered at depths of 10–15 cm, usually in discrete clumps.

Eggs and adults were predominantly found in this set of samples, but their contagious distribution pattern (Table 7) followed that of earlier samples of larvae (Table 2). Although samples were selected under badly damaged cane, many pits showed no evidence of white grub presence.

Sexing of adults found in the soil showed a higher proportion of males to females in *C. melolonthoides* (31 males: 11 females) but about equal in *B*. *werneri* (4 males: 5 females). The mean egg count for gravid females of *C. melolonthoides* recovered from soil was 11.2 with a range of 4–25.

**Table 7:** Summarized results of sampling for *Cochliotis melolonthoides* and *Brachylepis werneri* to assess phenology at JSP, 27 April–5 May 1987. Location LS10 are *B*. *werneri*; all the other locations are *C. melolonthoides*; other species were recorded in small numbers but are not presented here.

Location	No. of pits	Total volume (m^3^)	No with eggs	L_1_/m^3^	L_2_/ m^3^	L_3_/ m^3^	Adults/ m^3^
KN13	4	9.07	3	1.5	0.3	0.7	9.3
MN	7	41.51	0	0.0	0.0	0.0	0.5
KN6B	6	7.16	0	0.0	0.0	0.0	0.7
KN13	5	2.49	3	25.3	0.8	0.0	4.4
LS10	5	7.37	0	0.0	0.0	0.0	0.7

Both the larger white grub species (*C. melolonthoides* and *B*. *werneri*) seem to have a discrete one year cycle with some overlapping generations. For *C. melolonthoides* the life cycle probably follows this pattern: April–May (or at least the advent of main rains) a high proportion of adults emerge and mate, eggs are laid, first and second instar feed for 1–2 months on organic debris and small roots and then by September–October the effect of third instar feeding becomes apparent. The third instar lasts until January–February when pupation occurs. It is surmised that *B. werneri* follows a similar pattern except there was no clear evidence of the pattern of adult emergence, and third instar larvae were still found in May. 

### 3.7. Rearing Field Collected Larvae

The method was not satisfactory. The mosquito netting secured in the form of a cone frequently became dislodged due to high winds. This, combined with excessive water logging and ant infestation, meant there was little success in rearing larvae. Only one specimen was recovered in April, five months after the half drums were set up. This adult was found in the half drum set up with larvae of sp. C., and was identified as *Schizonycha* ?*vastatrix*. However, with only one adult reared, and the small chance of contamination of the half-drums, this association can only be considered provisional. One month after the half drums were set up, larvae of *B. werneri* were found to have been killed by an *Ophiocordyceps* fungus – see Section 3.11.

Using the small scale rearing method for bioassays 50% hatch was recorded in 10 days and first instar larvae were either preserved for future identification or used in development studies.

### 3.8. Alternative Hosts for Larvae

The results are presented in Table 8. After digging three replicates in each habitat, it became apparent that the numbers of white grub recovered were very low in both areas, and the exercise was abandoned.

**Table 8:** Pit samples (0.5 × 0.5 × 0.5 m) to compare white grub incidence under grass weeds and adjacent cane row in LS10/11, October 1986.

Site	Replicate	*Brachylepis werneri* L3	*Brachylepis werneri* L3 with *Ophiocordyceps barnesii*	Other white grubs
Grass weeds	1	0	0	1
	2	2	0	0
	3	1	1	0
	total	3	1	1
Cane row	1	0	1	0
	2	1	1	0
	3	0	0	0
	total	1	2	0

### 3.9. Extrapolating Population Density – Damage Relationships

The measurements of head capsule width and volume for the available instars of the six white grub species are given in Table 9.

**Table 9:** White grub head capsule width and body volume measurements.

Species		Head capsule width (mm)			Volume (ml)		
	Instar	Mean	SD	n	mean	SD^a^	n
*Cochliotis melolonthoides* (larva A)	3	7.85	0.32	10	2.47	0.63	10
	2	5.23	0.06	3	0.60	0.2	3
?*Triodontella* sp. A (larva B)	3	3.39	0.16	21	0.195	-	13
	2	2.11	0.13	14	0.063	-	8
	1	1.30	-	1	-	-	-
?*Schizonycha* sp. (larva C)	?3	4.36	0.35	7	0.775	0.31	6
Unknown Melolonthinae sp. (larva D)	3	2.50	0.19	11	0.092	-	6
	2	1.93	0.07	3	0.067	-	3
Unknown Cetoniinae sp. (larva E)	?2	1.90	0.20	3	-	-	-
*Brachylepis* *werneri* (larva F)	3	8.09	0.29	7	3.64	0.99	7

^a^ where none is given, the larvae where measured together and the average calculated.

**Figure 6:**
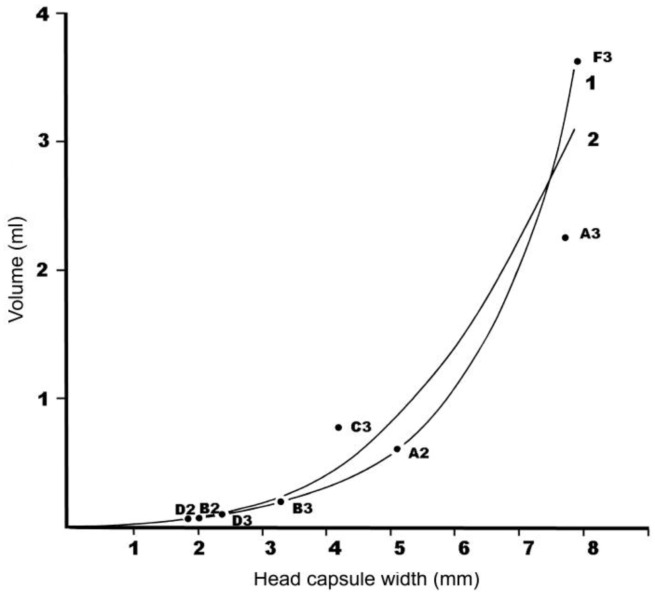
The relationship between larval volume and head capsule width. The fitted lines are: **1**, ln V = 0.646W−3.89 and **2**, V = 0.00606W^3^ −0.00484 where V = volume in ml and W= width of head capsule in mm. A–F = larva type followed by instar (A = *Cochliotis melolonthoides*; B = ?*Triodontella* sp. A; C = ?*Schizonycha* sp.; D = Unknown Melolonthinae sp.; F = *Brachylepis werneri*).

In Figure 6 the mean volume for each species instar is plotted against the mean head capsule width. Two equations were used to describe this relationship, both of which had a high correlation coefficient (r^2^ > 0.95, *p* < 0.001):

ln V = 0.646W − 3.89(1)

V = 0.00606W^3^ − 0.00484 (2)

where V = mean volume (in ml) per larva, and W = mean width of head capsule (in mm). Using these two equations, the volumes appropriate to each larva instar head capsule width were calculated (Table 10), and these were compared with a “standard larva” which was defined as the average volume of instar 3 *C. melolonthoides*. Finally, using Jepson's [7] figure for the economic threshold of 2–2.5 larvae of *C. melolonthoides* per stool, the extremes of the likely economic threshold for the species present at JSP were calculated, taking the lowest and highest results for the two models (Table 10).

**Table 10:** White grub volumes for final instars of different species calculated from fitted equations, their equivalence in terms of “standard larvae” (the mean volume of L3 *Cochliotis melolonthoides*), and indicative economic thresholds (the lowest and highest figures obtained using the two derived models).

Species	Using ln relationship		Using cube relationship		Indicative economic
	Volume	Standard larvae	Volume	Standard larvae	Threshold per stool
*Brachylepis werneri* (larva F)	3.75	0.9	3.20	0.9	1.7–2.3
*Cochliotis melolonthoides* (larva A)	3.22	1.0	2.92	1.0	2.0–2.5
?*Schizonycha* sp. (larva C)	0.347	9.3	0.498	5.9	12–23
?*Triodontella* sp. A (larva B)	0.189	17.0	0.237	12.3	25–43
Unknown Melolonthinae sp. (larva D)	0.104	31.0	0.089	32.8	62–82

### 3.10. Field Observations on Population Density – Damage Relationships

Table 11 gives the results for this test. Larvae were found both under cane which had no apparent symptoms of attack and under unhealthy cane. However, linear regression analysis showed that the number of larvae found increased significantly with the degree of damage recorded (*F*_1,28_ = 26.3, *p* < 0.05).

**Table 11:** Number of third instar *Cochliotis melolonthoides* recorded in standard 0.5 × 0.5 × 0.5 m quadrats in areas of block KN13 assessed with damage index of 1–5 (1 = all tillers are healthy; 2 = up to 1/4 of tillers are yellow and stunted; 3 = 1/4 to all tillers are yellow and stunted; 4 = all of stool stunted, some dead or dying tillers; and 5 = stools either completely dead or only a few green tillers which are small and unhealthy).

Location	Damage Index					
KN13/	1	2	3	4	5	Mean
2	0	5	1	10	12	5.6
2	2	4	6	12	20	8.8
7	1	1	9	5	12	5.6
7	2	3	7	35	36	16.6
8	7	1	19	37	53	23.4
8	3	12	19	41	50	25
Mean	2.5	4.3	10.2	23.3	30.5	14.2

### 3.11. Natural Enemies of White Grubs

#### 3.11.1. Field Observations

Predators were not commonly observed in the field, and no predation or larval remains that may have been killed by predators were recognized. Ants were found in the soil to 91 cm (36 inches), but since large numbers of white grubs were also present, cannot be efficient predators. Doubtless birds will take a large number of white grubs at plowing. Warthogs were observed to have dug in areas of high infestation, presumably locating the larvae by scent. The damage they cause by digging is considerably greater than that caused by the white grubs.

No evidence of ectoparasitism was found on either visit. In October 1986, 42 third instar larvae of *C. melolonthoides* from KN06A and 75 from KN13 were dissected; no evidence of parasitism was found. In April–May 1987, over 3,000 adult *C. melolonthoides* were dissected to assess reproductive status (Table 6); no evidence of internal parasitism was found, although only large parasitoid larvae would have been noticed in this work.

A small number of ambiguous finds when sieving soil may have reflected the presence of parasitoids. Digging in LS10/09 in October 1986, two large, empty, yellow-brown cocoons were found. These resemble scoliid cocoons examined in the Natural History Museum, London by MJWC, and could have been of a *Campsomeris* sp. One was at 76–84 cm (30–33 inches) depth and quite fresh, the other at 30–38 cm (12–15 inches) was rather older. Subsequently in LS10/11 several much smaller cocoons were found measuring 15 mm, dark brown in color and wider at one end than the other. These contained yellow prepupae, but all were damaged in collection. They were found at a depth of 46–61 cm (18–24 inches) where, following recent rain, the soil was waterlogged. Possibly these cocoons represent one of the smaller *Campsomeris* spp. which may have attacked second instar larva of *B*. *werneri*, which was much the commonest white grub in the block. No evidence suggesting the presence of *Campsomeris* spp. was found in May 1987.

In the vicinity of LS10, specimens of a large bombyliid (bee fly) were observed on two days. One was caught and subsequently identified as *Ligyra enderleini* Paramanov. Given the known habits of related bombyliids, the large size of this species, and the range of hosts available, this species is quite likely to be a hyperparasitoid of a large scoliid such as that which formed the large cocoons found in the same area.

None of these observations were clear cut, but they do indicate that if present at this time, parasitoids were scarce and having little impact on the white grub populations.

The only evidence of pathogens was of *Ophiocordyceps barnesii* which was associated with mummified third instar larvae of *B*. *werneri* in LS10 and adjacent areas. In addition to field collections between blocks LS10 and LS11 and an adjacent grassy area (Table 8), the fungus was found in one of the rearing trials. Half drums 1 and 2 set up with ten third instar *B*. *werneri* each (Table 8) was found to contain mummified larvae with *Ophiocordyceps* stroma (spore producing bodies) after one month. The species has since been confirmed to be *O. barnesii*, a widespread pathogen of Scarabaeidae larvae [21].

In October 1986, *O. barnesii* was found killing third instar larvae of *B*. *werneri* in LS10. Incidence varied from 0 out of 9 in LS10/09, to 6 out of 28 in LS10/10, to 2 out of 5 in LS10/11 (*i.e.*, 0%–40%). The more detailed survey in May 1987 is reported below. The fungus fills the body cavity of the host larva with a solid mass of white hyphae; the corpse then desiccates and becomes “mummified”, brown and hard to the touch. Then from the head capsule (which in all observed cases was uppermost on the mummy) a stroma (or “shoot”) grows up to the soil surface (the mummy was always in the top 10 cm of soil) where the tip of the shoot sporulates. Since some mummies were very fresh—one had not even turned brown, it is likely that more larvae would have succumbed as the rainy season progressed, so that the mortality rates suggested above are probably underestimates.

#### 3.11.2. Survey for *Ophiocordyceps barnesii*

In May 1987, when this survey was carried out, infected specimens were usually found with 1–2 bifurcate stromata branches with orange tips emerging just above the soil surface. The healthy larvae were found 5–10 cm below the soil surface, whilst adults were generally found directly under the cane stool. The specimens were found in fine loamy soils, pH8, under the cane trash or at the base of the cane stool usually in a shaded area. On one occasion four specimens were found in a 5 cm radius. The details of the sample pits and results are given in Table 12. Eleven mummified and 29 healthy third instar larvae of *B*. *werneri* were found, *i.e.*, 27.5% were killed by *O. barnesii*.

**Table 12:** Sampling for *Brachylepis werneri* and *Ophiocordyceps barnesii*, May 1987.

Location	Pit size length x breadth x depth (cm)	Apparently healthy *Brachylepis werneri*					L_3_ *Ophiocordyceps barnesii* mummy
		L_1_	L_2_	L_3_	Pupae	Adults	
LS10/09	285 × 85 × 35	0	0	1	0	0	1
	280 × 30 × 24	0	0	2	0	0	7
	203 × 70 × 20	0	0	3	0	0	0
	110 × l00 × 40	0	0	4	0	0	0
	l20 × 90 × 40	0	0	3	0	0	0
	260 × 90 × 30	0	0	6	0	1	0
	103 × 83 × 20	0	0	4	0	0	1
LS10/08	70 × 24 × 10	0	0	2	0	0	0
	8l × 22 × 16	0	1	0	0	0	0
	103 × 21 × 11	0	0	0	0	0	1
	80 × 20 × 15	0	0	1	0	0	0
	200 × l00 × 50	0	0	3	0	0	1
	50 × 38 × 20	0	0	0	0	0	0
Total		0	1	29	0	1	11

#### 3.11.3. Sampling for Entomopathogenic Nematodes (EPNs)

Of the first instar larvae of *C. melolonthoides* set up with soil from the field, one brick red larva was recovered with nematodes visible beneath the cuticle one week later. The five third instar *B*. *werneri* larvae then added to the soil sample were all dead within three days. Of these, three had positive symptoms of EPNs; two were taken to UK for identification and multiplication and one was left at JSP for further tests. The remaining two larvae had no external symptoms but showed red pigmentation in their internal “bacterial soup” contents. This EPN was subsequently identified as a *Heterorhabditis* sp. The symbiotic bacterium was isolated in the UK and its insecticidal properties demonstrated. This nematode, being indigenous to Somalia and therefore likely to be heat tolerant, may be a useful management tool in the future.

## 4. Discussion and Conclusions

During two brief visits to Somalia, we have accumulated information on the biology, phenology, pest incidence and management options for white grubs in south-west Somalia. Given the time limitations, much of this information is of a preliminary nature, but is sufficient to give clear indications as to which are the economically damaging species, their biology and phenology. Useful indications have been documented regarding sampling methods and economic thresholds. Biological control options have been identified but would need further development and testing. However, bearing in mind that these observations were made 25 years ago, some conclusions are still valid, but others, particularly those relating to scarabaeid incidence and damage, can only be considered snapshots from the past, and new studies would be needed to assess the current situation.

The results show that a large proportion of the larvae found in the transect would have been missed in a standard quadrat: 42, 55, 38 and 52% respectively for samples 1–4 (Figure 5). If the quadrat size was changed to 1 × 0.5 × 0.5 m across the cane row the percentage missed would be reduced to 10, 21, 31 and 30 for the four holes. If, however only larvae of *C. melolonthoides* and *B*. *werneri* are considered, the standard quadrat would have missed 30, 50, 25 and 17% while a 1m wide quadrat would only miss 10, 17, 0 and 0% respectively. However, it needs to be asked, how reliable are the records of distance from the center of the cane row? As suggested above, there was undoubtedly some displacement of the larvae during excavation, but overall this should have evened out, and in any event many larvae were discovered in situ including almost all large individuals (*C. melolonthoides* and *B*. *werneri*). Hence, the recorded position of individual larvae may be in error but the pattern is probably reliable. This could be confirmed by excavating additional transects using Jepson's [7] method in which an initial trench was dug in the inter row parallel to the cane row, and then extended 7.5 cm (3 inches) at a time across the row. In this way a reliable measure of the distance of each larva from the cane row would be obtained (at the cost of potential inaccuracies in the depth measurements). If, in future, absolute measures of population density are needed, it would be appropriate to use a quadrat at least 1 m wide across the cane row. However, the advantage of smaller quadrat size is clear for logistic reasons, and this is still appropriate, providing it is recognized that a proportion of the white grub larvae will be missed as they are further from the middle of the cane row than the quadrat extends. Further sampling would be needed to assess whether some consistent conversion factors could be derived and applied. 

As Carnegie and Leslie [37] concluded in South Africa, the use of a light trap provides a cost effective way of monitoring adult populations of those Scarabaeidae species which fly by night. Although there may be white grubs which do not fly by night, there is no evidence that any of these occur in JSP, since the same species dug from sugar cane fields as adults were also caught in the light trap. Using a light trap also provides a very efficient method of collecting specimens for identification and taxonomic studies. For example in KN06A, a day's digging of 2 m^3^ produced 2 adult *Triodontella* sp. A; the next night the light trap caught 1,023 at the same place.

Based on the results obtained, it seems that *C. melolonthoides* and perhaps *B*. *werneri* (the two larger species) have an annual life cycle with the adults flying at the beginning of the main rains only. In contrast, at least some of the smaller species fly in both rains, although we cannot say whether this represents overlapping generations or two generations per year linked to the bimodal rains.

The method adopted here to extrapolate population density – damage relationships is far from precise and includes several untested assumptions, but it provides an approach to assess the relative damage that different sized white grubs may do to sugar cane. The indication that white grubs of the smaller species (e.g., ?*Triodontella* sp. A (larva B) and unknown Melolonthinae sp. (larva D)) can be tolerated at densities 10–30 times those of the two large species (*C. melolonthoides* and *B.werneri*) may be a useful guide.

Comparing the larval densities recorded at JSP with these economic thresholds, the only species likely to locally exceed the economic threshold at the time these observations were made are *C. melolonthoides*, *B*. *werneri* and possibly *Schizonycha* spp. Although sometimes more common, the smaller larvae of ?*Triodontella* sp. A (larva B), unknown Melolonthinae sp. (larva D) and unknown Cetoniinae sp, (larva E) did not reach their estimated economic thresholds.

This has implications for the most efficient survey strategy. If *C. melolonthoides* and *B*. *werneri* have annual life cycles, and October (and perhaps a few months following) is the period when they are full grown, then survey work should concentrate on this period when the only species of economic importance are present as large larvae and hence immediately distinguishable from small unimportant species.

Although the sample size was comparatively small and should be repeated in other fields with different soil types, the correlation of white grub population density with the damage index showed promise as a way to simply and quickly assess white grub populations and damage without the major effort of comprehensive soil sampling, although spot sampling should still be anticipated to confirm which species is present. For *C. melolonthoides* soil sampling would be restricted to the season when third instar larvae are present, October–December. 

Several natural enemies of *Cochliotis melolonthoides* have been reported from Tanzania [7], but no evidence was found for natural enemies attacking this species at JSP. For example, although *Ophiocordyceps barnesii* has been reported as a sometimes common pathogen of *Cochliotis melolonthoides* in Tanzania [9,20,21], in our studies we found it affecting *B*. *werneri* only, even though *C. melolonthoides* was considerably more common in nearby blocks. Cross infectivity trials could not be attempted in May 1987 as inadequate numbers of third instar *C. melolonthoides* larvae were available. In the rearing trails in half drums, set up in October 1986 using soil from white grub-free blocks, only *B*. *werneri* became infected with *O. barnesii*. However, this provides only circumstantial evidence as most larvae died anyway, there were no trials with *C. melolonthoides* and the *B*. *werneri* larvae may well have been infected before they were set up.

The EPNs and *O. barnesii* found in our surveys are both considered to have potential as biopesticides, if interventions were needed to reduce populations of white grubs. EPNs can be easily mass produced and are widely tested and used for the control of pests in restricted, moist places, including soil pests. *Ophiocordyceps* fungi cannot be mass produced at this time, so the immediately available option would be to field-collect the sporing bodies and redistribute the spores to areas where white grub damage occurs, but the fungus does not seem to be common.

The white grub incidence within individual fields is very heterogeneous, and outbreaks had been very localized. We considered to what extent such a pattern can be linked to physical factors such as soil type, cane variety, levels of irrigation, presence of alternative hosts *etc*. We concluded that the data to answer this question are not available, and extensive and prolonged surveys would be necessary to find any correlations and manipulative experiments to then demonstrate cause and effect

At the present time, although Somalia depends heavily on agriculture to sustain its economy [38], the large scale growing of sugar cane is not being practiced due to the on-going state of unrest. This is starting to change, so that in the future, and it may be that sugar cane production will be developed again, in which case, these observations will be relevant to that future production system. On the other hand, other priorities may arise, so that food crops or other agricultural land-use is developed in south-west Somalia. However, given that white grubs are polyphagous, then many of the food crops likely to be grown will also be affected, so again these observations will be relevant. Finally, the observations, as they relate to crops grown under irrigation in an area of weak, irregular, bimodal rainfall, may be relevant to other parts of Africa, either now, or in the future as a result of climate change. Thus, although not immediately applicable in Somalia, we believe these observations and conclusions will be useful in the future.
